# Bullous Pyoderma Gangrenosum in a Patient with Acute Myelogenous Leukemia as a Pathergic Reaction after Bone Marrow Biopsy

**DOI:** 10.4274/tjh.2016.0096

**Published:** 2017-12-01

**Authors:** Nur Efe İris, Reyhan Diz-Küçükkaya, Mutlu Arat, Zahide Eriş

**Affiliations:** 1 İstanbul Bilim University Faculty of Medicine, Department of Infectious Diseases and Clinical Microbiology, İstanbul, Turkey; 2 Avrupa Florence Nightingale Hospital, Clinic of Infectious Diseases and Clinical Microbiology, İstanbul, Turkey; 3 İstanbul Bilim University Faculty of Medicine, Department of Internal Medicine Division of Hematology, İstanbul, Turkey; 4 İstanbul Bilim University Faculty of Medicine, Department of Dermatology, İstanbul, Turkey

**Keywords:** Acute myelogenous leukemia, Bullous pyoderma gangrenosum, Pathergy

A 59-year-old male patient presented with a wound over the sacral region on a bone marrow biopsy puncture that had been present for 3 weeks (Figure 1). There was an ulceration of 6x4 cm with a bullous margin. Bullous pyoderma gangrenosum (PG) was diagnosed by the dermatology consultant. Histopathologic examination of the biopsy specimen from the ulcer showed necrosis with an underlying mixed inflammatory cell infiltration within the dermis extending to the subcutis. Cultures of skin biopsies were negative for bacteria, fungi, and atypical mycobacteria. A bone marrow biopsy showed acute myelogenous leukemia (AML) transformed from myelodysplastic syndrome.

PG is an uncommon neutrophilic ulcerative skin disease. In contrast to its name, PG is neither an infectious nor a gangrenous condition. Pathergy is commonly observed, especially after debridement of a lesion [1,2]. In PG there is an excessive inflammatory reaction to trauma of the skin by a needle. In this case there was a pathergic reaction after bone marrow biopsy.

Definitive diagnosis requires both clinical recognition and exclusion of infectious or neoplastic disorders [3]. PG is usually associated with an underlying systemic disease [1,4]. Based on clinical morphology, PG is classified into four variants: ulcerative, pustular, bullous, and vegetative [5]. Bullous PG is commonly associated with myeloproliferative diseases [5]. Association with leukemia signifies a poor prognosis [5].

Our patient was in remission for AML, he underwent allogeneic hematopoietic stem cell transplantation, and the PG resolved completely.

## Figures and Tables

**Figure 1:**
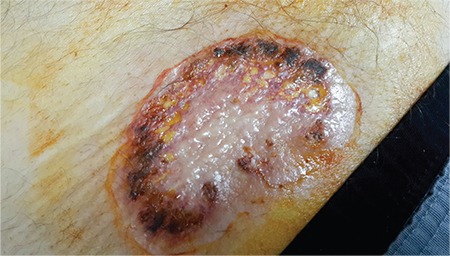
Bone marrow biopsy puncture area with 6x4 cm cribriform ulceration with expanding bullous margin.
